# Progressive Medicine, Volume II

**Published:** 1900-01

**Authors:** 


PROGRESSIVE MEDICINE, VOLUME II.—A Quarterly Digest of Advances, Discoveries, and Improvements in the Medical and Surgical Sciences. Edited by Hobart Amory Hare, M. D., Professor of Therapeutics and Materia Medica in the Jefferson Medical College of Philadelphia. Octavo, handsomely bound in cloth, 472 pages, 56 illustrations and 3 full-page plates. Lea Brothers & Co., Philadelphia and New York.

This volume presents carefully prepared and exhaustive papers upon the following subjects:

"Surgery of the Abdomen, Including Hernia." By Wm. B. Coley, M. D., of New York City.

"Gynecology." By John G. Clark, M. D., of Philadelphia.

"Diseases of the Blood, Diathetic and Metabolic Disorders, Diseases of the Spleen, Thyroid Gland and Lymphatic System." By Alfred Stengel, M. D., of Philadelphia.

"Ophthalmology." By Edward Jackson, M. D., of Denver.

The physician who wants to keep up with medical progress should take these books which appear quarterly. The subjects discussed are sufficiently lengthened out to give a comprehensive view, and at the same time they are brief enough to require but a short time in which to read them. Men are chosen to discuss the various subjects who, by their professional training, are best able to reflect the latest and best digested truths of medical progress, thereby stamping all statements with expert authority. Thus, one may have each quarter a comprehensive and an authoritative review of scientific medicine, in convenient form, and at a nominal cost.

